# Development and Characterization of Carbon-Based Adsorbents Derived from Agricultural Wastes and Their Effectiveness in Adsorption of Heavy Metals in Waste Water

**DOI:** 10.1155/2022/1659855

**Published:** 2022-09-23

**Authors:** L. Natrayan, S. Kaliappan, C. Naga Dheeraj Kumar Reddy, M. Karthick, N. S. Sivakumar, Pravin P Patil, S. Sekar, Subash Thanappan

**Affiliations:** ^1^Department of Mechanical Engineering, Saveetha School of Engineering SIMATS, Chennai 602105, Tamil Nadu, India; ^2^Department of Mechanical Engineering, Velammal Institute of Technology, Chennai-601204, Tamil Nadu, India; ^3^Department of Civil Engineering, Aditya College of Engineering and Technology, Surampalem, India; ^4^Department of Mechanical Engineering, Velammal Engineering College, Velammal New-Gen Park, Ambattur-Redhills Road, Chennai 600066, Tamil Nadu, India; ^5^Department of Mechatronics Engineering, TISHK International University, Erbil, Iraq; ^6^Department of Mechanical Engineering, Graphic Era Deemed to Be University, Bell Road, Clement Town, 248002 Dehradun, Uttarakhand, India; ^7^Department of Mechanical Engineering, Rajalakshmi Engineering College, Rajalakshmi Nagar Thandalam, Chennai 602 105, Tamil Nadu, India; ^8^Department of Civil Engineering, Ambo University, Ambo, Ethiopia

## Abstract

The current work focuses on peanut shells and agricultural wastes richly in many nations subjected to pyrolysis treatment at various temperatures in the range of 500–800°C to determine the feasible physiochemical characteristics of the biochar. The biochars with the high surface area were employed to adsorb Pb^2+^ (lead) ions, the heaviest pollutants in the water bodies. The raw material, biochar, and pyrolyzed biochar were characterized by SEM, FTIR, partial and elemental analysis, and BET tests. The adsorption characteristics of the biochar, pre- and postpyrolysis treatment, were studied with the assistance of batch adsorption tests under varying test conditions. Adsorbing conditions were determined by evaluating the effects of adsorbing parameters like initial concentration of the lead in water, pH of the adsorbent, contact time, and mixing speed on the effective adsorption of Pb^2+^ ions from water. Langmuir, Freundlich, and Themkin isotherm expressions were employed to study the experimental results. The adsorption kinetics study showed that the synthesized biochars were chemically stable enough to adsorb the Pb ions onto the surface.

## 1. Introduction

The rapid growth of the population in tandem with the technology and growing demands of the population has resulted in the increased waste generation of various kinds and varieties. The wastes alter the physical, chemical, and biological properties of the soil and water bodies, causing them to be unconsumable for agricultural and day-to-day activities [1]. The most critical issue is that the cause of water pollution is industrial wastewater with heavy metal concentration [2]. The most critical heavy metals in industrial effluent are chromium, arsenic, lead, and so on. Lead, one of the heaviest metals observed in industrial effluent, is let out by mines, petroleum refineries, paint and battery manufacturing units, electronic units, and glass production [3]. It has considered the benefits of using lead in various industries that need effective removal of lead from industrial wastewater because of its superior mechanical, physical, and chemical characteristics. Composites as a homogeneous and heterogeneous mix of different types of known adsorbents have been developed to improve performance synergistically.

Lately, researchers have conducted superior techniques for treating industrial effluents intended for heavy metal adsorption and reducing their drastic impact on the environment. However, most techniques employed for this dedicated process are not cost-effective and possess complex operating procedures. Therefore, the need for simple yet high-performance treatment procedures was stressed by the researchers for the removal of heavy metals from the industrial effluent. Adsorption is a cost-effective, simple technique that produces a high yield in the removal of heavy metals from industrial effluent [4]. Recently, the use of agricultural wastes that contain carbon has seen increased usage for the adsorption of heavy metals from industrial effluent. Various biological wastes have been chemically or physically activated to perform the role of adsorbent. One such activation process is applying heat and pyrolysis [5]. Pyrolysis is a heating process that occurs in anaerobic conditions to produce increased carbon content, activated carbon, or biochar [6]. The pyrolysis product's production depends on parameters like period and reactor temperature. These two process parameters vary to obtain the products with the desired properties [7]. Solid support immobilized organic compounds are attracting great interest from researchers and are categorized based on the nature of the solid substrate. These include synthetic organic polymers, natural biomacromolecules (cellulose, chitosan), and inorganic oxide species. Many literatures have reported on producing activated carbon from agricultural wastes and its employability as an adsorbing agent in industrial effluent processing [8]. Activated carbon from various agricultural wastes like sugarcane bagasse, pine cones [9], tea waste [10], apricot shells [11], doum palm shells [12] peach stones [13], Lapsi seed stone, and saffron leaves, and so on. [14]. The reason for selecting carbon-based adsorbents is credited to the increased adsorption capacity, good surface properties, and uniform adsorption. Moreover, the carbon-based adsorbent is one of the most cost-effective solutions for removing heavy metals from industrial effluents.

The novelty of this research work is its aim to investigate the absorptivity of biochars manufactured by pyrolyzing the peanut shells for the adsorption of Pb^2+^ ions from water. The initial experiments involved finding the surface and chemical properties of industrial wastewater (biomass) and biochar. The initial set of experiments was followed by batch testing, varying the test conditions like concentration of Pb^2+^ ions, adsorbent quantity, temperature, contact time, pH, and mixing speed. The study concludes with the adsorption isotherms for Pb^2+^ ions.

## 2. Materials and Methods

The agricultural wastes, especially the peanut shells, were collected from the local agricultural store and subjected to a temperature of 85°C for drying purposes for 12 hr. The dried specimens were cut by a stainless steel blade and sieved to a dimension below 200 *μ*m to reduce the mass and heat transfer during the experimentation [15]. The batch adsorption tests were conducted by preparing standardized solutions of lead nitrate, Pb (NO_3_)_2_ [16].

### 2.1. Raw Material Characterization

#### 2.1.1. Ultimate and Proximate Analysis

The CHNS-932 model analyzer was employed to perform the ultimate analysis, where C, H, S, and N are simultaneously measured. The E2K oxygen bomb calorimeter was employed to quantify the calorific value of the peanut shell [17]. The Protherm PLF120/5 ash furnace was employed to conduct the proximate analysis. To determine the volatile, moisture, and ash content in the raw agricultural wastes, the specimens were kept in the preheated ash furnace at a temperature of 1000°C for 15 min, 900°C for 90 min, and 85°C for one day, respectively [18].

### 2.2. FTIR Analysis

A Perkin Elmer FTIR spectrometer was employed to measure the FTIR spectra of the peanut shells in the spectral range of 300 to 5000/cm with the assistance of potassium bromide and KBr pellets [19]. With the assistance of the peaks presented by the functional groups, the structures of the biomasses were found.

### 2.3. Pyrolysis

The 30 g specimen of peanut shells is electrically heated at varying temperatures in the reactor range of 500–800°C, with the volume of a fixed bed of 500 cm^3^. The flow rate of N_2_ gas was maintained at 80 mL/min during the pyrolysis with a heating rate of 30°C/min [20]. The heat couple provided the heat energy to the pyrolysis reactor, which was placed in the interior of the reactor [21]. Post the completion of the heating process, the specimens were kept at a constant temperature for 1 hr to complete the pyrolysis process [22].

### 2.4. Biochars Characterization

The N_2_ adsorption isotherms provided the information for the BET analysis. The characterization was done at 77 K in the 3Flex Micro Metrics Surface analyzer. The specimens were degreased at 250°C for 12 hr in a vacuum beforehand to the gas adsorption tests [23]. The relative pressure values provided the data on the adsorption. The BET surface area was computed from the N_2_ adsorption isotherms with the assistance of the BET expression (Brunauer–Emmett–Teller) [24].

### 2.5. Batch Adsorption Tests

**Figure 1:**
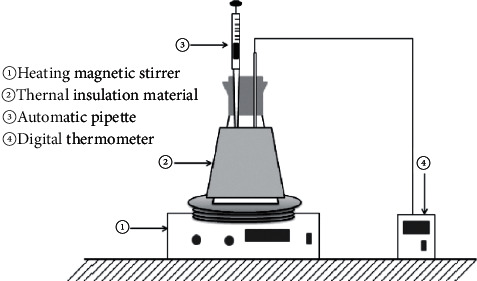
Schematic diagram of the batch adsorption experiment.

The adsorption efficiency of the peanut shell from the diluted solution was determined through batch adsorption tests [25]. The experimental setup is displayed in Figure 1. The lead nitrate, Pb (NO_3_)_2_ of 1 L, was diluted by mixing it with the distilled (DI) water to get the desired concentration for the batch adsorption tests. 300 mL of lead-contaminated solution was employed for the batch adsorption equilibrium experiments [26].

Conditions that influenced the adsorption process like solution pH varied from 2 to 5, the dosage of the adsorbent varied from 2 to 4 g/L, the initial Pb (NO_3_)_2_ concentration varied from 20 to 50 ppm, and the contact time was done with the maximum limit of 200 min [27], the temperature during the process was varied from 30 to 50°C, and the stirring speed was varied from 300 to 600 RPM. 0.1 M hydrochloric acid, HCl, and 15% weight/volume of Azane, NH_3_ solution were used to adjust the pH of the solution. By filtration, the samples were collected at preset intervals [28]. The filtrate product was analyzed to determine the concentration of Pb^2+^ ion residue by Perkin Elmer equipment. The quantity of Pb^2+^ions adsorbed per unit mass of the adsorbent (q_e_) was estimated based on the collected data using (1), and the adsorption yield was computed using equation (2). Therefore, the best adsorbing conditions of Pb^2+^ were determined.

(1) $$\begin{array}{c} q_{e}=\frac{\left(C_{o}-C_{e}\right)*V}{M}, \end{array}$$

(2) $$\begin{array}{c} {\text{Pb}}^{2+}\text{ion adsorption }\%=\frac{\left(C_{o}-C_{e}\right)}{C_{o}}*100, \end{array}$$

where *q*_*e*_ is the quantity of Pb^2+^ ions adsorbed per unit gram of the adsorbing agent (mg/g), *C*_*o*_ and *C*_*e*_*V* is the volume of the contaminant-containing solution, *L*, and m is the weight of the adsorbing agent. Initial and final concentrations of Pb^2+^ ions in the solution are given as ppm, respectively. All adsorption tests were done five times to verify repeatability, and the average values were utilised in data analysis [29]. The relative standard deviations were determined to be within 1%.

## 3. Results and Discussion

### 3.1. Characterization of the Peanut Shell

The agricultural waste and peanut shells were characterized for the proximate test, calorific quantity, and ultimate analysis. The peanut shell characterization results in Table 1 show that peanut shells have a great potential application for heavy metal removal in industrial effluent by pyrolysis, as this biomass contains less than 10% [30]. The other important parameter in biomass is its volatile content percentage. The volatile content in the biomass generally gets evaporated during the pyrolysis stage. The volatile content in the peanut shell biomass was found to be 77%, per the literature [31].

The ash content of the peanut shell biomass was measured to be 1.95%. According to the literature [32], the lower the ash content in the biomass, the higher the adsorption characteristics. The chemical analysis in Table 1 shows that the percentages of sulfur and nitrogen are 1.51% and 1.18%, respectively. Since the percentages of these chemicals are very low, the possibility of the formation of the corroding environment in the pyrolysis chamber is very low [33]. The heating value of the peanut shell was measured to be 16 MJ/kg. The higher heating value of the peanut shell shows that the energy content can increase during pyrolysis and also assists in releasing pyrolysis oil during the pyrolysis process [34].

**Figure 2:**
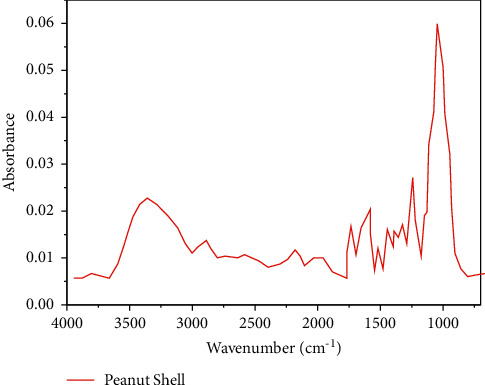
FTIR Spectrum for peanut shell.

The FTIR spectrum of the peanut shell is presented in Figure 2. The wide vibration curves are observed in the range of 3485–3896/cm wavelength, which shows the hydroxyl (OH) presence. The OH presence is credited to the water content in the peanut shell [35]. The stretching of CH is observed in 2912–2968/cm and confirms the presence of aliphatic hydrocarbon [36]. The stretching at 1625/cm indicates the aldehyde presence, whereas the stretching vibrations at 1022/cm indicate the COH group, which includes alcohol, phenol, and ether.

### 3.2. Biochars Characterization

The BET analysis was used to compute the specific surface area of the pyrolyzed peanut shells. The pyrolysis of the biochar changes the surface area and absorptivity. The BET analysis concerning the temperature is displayed in Table 2.

From Table 2, it is observed that the surface area was observed to increase with the increase in the pyrolyzing temperature. At a higher temperature of 800°C, the surface area falls. The increase in the surface area is credited to the formation of cross-linking chemical agents. In contrast, the fall in the surface area is credited to the collapse of these structures at high pyrolyzing temperatures [37]. The rise in the temperature from 500 to 700°C led to a rise in the surface area. The higher the surface area, the better the adsorption results; therefore, the specimen pyrolyzed at 700°C is considered for adsorption study.

### 3.3. Effect of pH on the Adsorption of Pb^2+^ions

**Figure 3:**
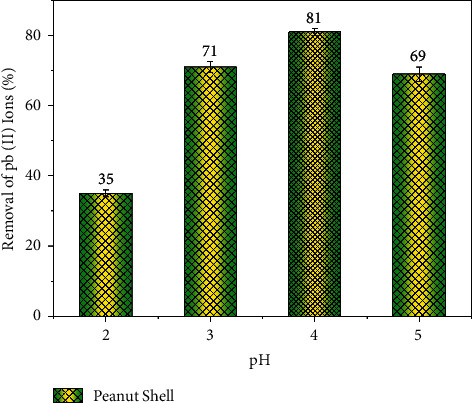
Effect of pH on adsorption.

The chemical content of the base solution, with the heavy metal content in a basic or acidic zone in terms of pH, is a key for the adsorption studies. The acid content of the initial solution tends to affect the adsorption rate as the acidity influences the surface charge [38]. When the solution is based on pH, the heavy metal residues react with the OH ions in the atmosphere [39]. As a result, the adsorption study was done in the acidic pH range of 2 to 5, with the dosage of the adsorbent varying from 2 to 4 g/L, the initial Pb (NO_3_)_2_ concentration was varied from 20 to 50 ppm, and the contact time was done with the maximum limit of 250 min, the temperature during the process was varied from 30 to 50°C, and the stirring speed was varied from 300 to 600 RPM [40]. The adsorption rate of Pb^2+^ions is presented in Figure 3. The adsorption rate in Figure 3 shows that the adsorption of Pb^2+^ions heavily influences the pH of the solution, where the hydroxyl ions have a key role compared to the lead ions in the base solution. At low pH, the dominant OH ions in the solution were more attracted to the active zones on the adsorbent's surface than Pb^2+^ ions, which possess a positive charge [41].

Therefore, the absorbent surface formed a positive charge with low electrostatic force between the surface and Pb^2+^ ions. However, the rise in the quantity of negative OH ions in the solution through the rise in pH leads to a high affinity between negative OH ions on the adsorbing surface and positive Pb ions [42]. Furthermore, poor hydraulic conductivity is not favoured as a packing medium. Hence, the utilization of oxide-coated sand has been found to be worth developing for the removal of heavy metals from the aqueous phase. Such an interaction led to increased adsorption of Pb ions [43]. This phenomenon was observed till the pH of 4; however, the precipitation of Pb hydroxide was observed at higher pH. This phenomenon was also observed in the investigation reported by Kaya et al. 2020 [1].

### 3.4. Effect of Temperature on Adsorption

**Figure 4:**
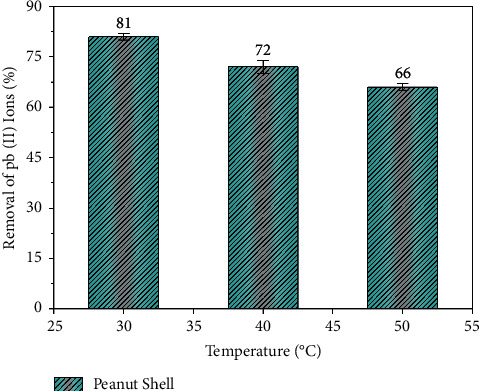
Adsorption rate of Pb ions concerning temperature.

The physisorption usually takes place at low temperatures due to the influence of van der Waals forces, which tends to decrease with the increase in the temperature. However, the chemisorption process, which is dependent on the chemical connections between the surface and the adsorbate, is affected by the heat, which is either exothermic or endothermic. The rise generally influences the chemisorption at the temperature [44]. The adsorption rate of Pb ions concerning the temperature is presented in Figure 4.

From Figure 4, it is observed that the adsorption of Pb ions increased with the increase in the temperature of the solution. Such a phenomenon is explained by the reduction of the activation energy with the temperature rise to improve the heavy metal adsorption [45]. Therefore, this phenomenon suggests that the process is endothermic. Evaluation of sand and oxide-coated sand for adsorptive removal of various heavy metals from aqueous solutions has been carried out [46].

### 3.5. Effect of Initial Lead Concentration

**Figure 5:**
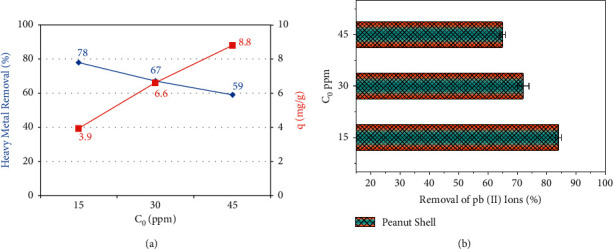
(a) Adsorption performance for varying Pb concentration. (b) Adsorption rate of peanut shell.

In general, the initial concentration of the test specimen plays a crucial role in the chemical exchange between the adsorbate and the adsorbent. The rise in concentration leads to sufficient mass transfer in the chemical reaction to increase the adsorption rate. However, there is only a restricted quantity of active centres to which ions can be adsorbed at the adsorbing agent's surface. The assumption is that the chances of ionic interaction with the restricted quantity of binding zones of the adsorbing agent's surface decrease with a rise in concentration. The adsorption rate of the peanut shell with respect to the variations in the initial concentration of lead is presented in Figure 5. Because the concentration is a critical driving agent for mass transfer between the contaminated solution and the peanut shell biochars to overcome resistance, a rise in the concentration resulted in a rise in the equilibrium absorbability. As a result, satisfactory adsorption is attained with low adsorption rates at low concentrations [47].

Figure 5(b) shows that the adsorption rate of the peanut decreased with the increase in the concentration of Pb ions. The increased adsorption at a reduced concentration of the Pb ions can be credited to the adsorbate molecule interactions with the binding zone of the adsorbent surface [48].

### 3.6. Effect of Dosage of the Adsorbent

**Figure 6:**
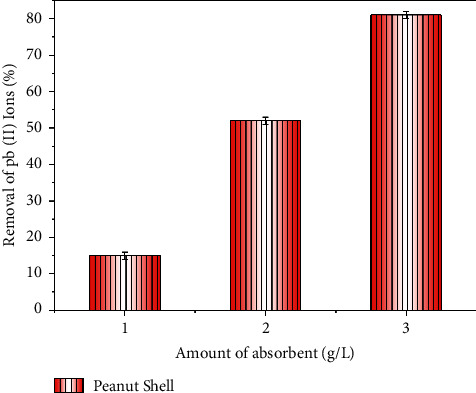
Influence of adsorbent dosage on Pb ions adsorption.

The increase in the adsorbing agent dosage results in increased contact with the adsorbing surface due to the rise in the chemical interactions with the active zones until a certain threshold where the adsorption rate rises [49]. The influence of adsorbent dosage in the adsorption of Pb ions by peanut shell biochars is presented in Figure 6.

Sand, traditionally employed as a filter medium for removing essentially suspended matter, has also been tried as an adsorbent for removing dissolved metal ions. Figure 6 shows that the rising of the adsorbent dosage increases the adsorption capacity by a higher removal of Pb ions. The maximum adsorption was observed at 4 g/L, which was credited to the rise in the chemical contact area between the adsorbate and the adsorbent [50].

### 3.7. Influence of Mixing Speed

**Figure 7:**
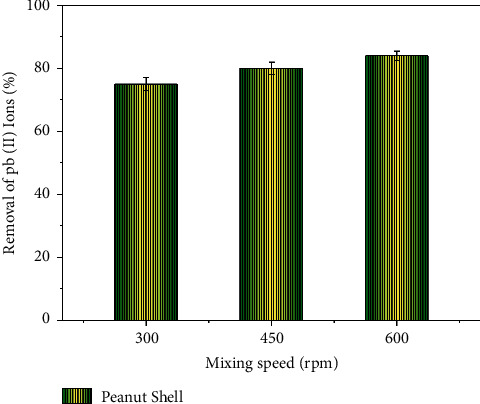
Influence of mixing speed on adsorption.

The mixing speed is reported to influence the dispersion of the adsorbing agent in the specimen solution in a homogeneous and effective mode to affect the adsorption capacity. The effect of the variations in the mixing speed on the adsorption of Pb ions is presented in Figure 7. From Figure 7, it is observed that the rise in the mixing speed resulted in increased adsorption of Pb ions as the increased stirring was observed to deteriorate the thin and fixed film which is present around the adsorbent, which also resulted in the formation of higher activation zones for the increased chemical interaction [51].

The use of high-speed stirring at 600 RPM produced a nonsignificant effect on the adsorption of Pb ions compared to low-speed stirring; such a phenomenon was also observed in Kaya et al. 2020 [1].

### 3.8. Influence of Contact Time on Adsorption

The adsorption process exists because of a dynamic equilibrium between the dissolved solvent in the solution and the dissolved solvent on the adsorbent surface. As a result, the effect of contact time on adsorption is determined by finding the concentration of the Pb in the solution. The influence of contact time on the adsorption capacity is presented in Table 3. Table 3 shows that the Pb ion concentration increases with the increase in the equilibrium duration. This phenomenon was observed till saturation was attained during the chemical interaction. Once the saturation process occurs, the chances of intraparticle diffusion are also possible [52]. This phenomenon occurs due to the presence of higher active zones for the increased surface adsorptions until the attainment of chemical balance to indicate the arrival of the chemical equilibrium. The arrival of chemical equilibrium reduces the adsorption capacity due to the reduction in the number of active zones on the adsorbent surface [53]. Faster adsorption at low time periods to attain the chemical equilibrium indicates the adsorbent's effectiveness in removing the Pb ions from water.

## 4. Adsorption Isotherms

The adsorption process at constant temperature is quantified with the assistance of adsorption isotherms. The adsorption isotherms indicate the distribution of molecules that are adsorbed into solid and liquid phases on the arrival of the chemical equilibrium. The experimental results are employed to determine the optimal adsorption isotherm, which describes the adsorption process. The usefulness of the isotherm equations in adsorption investigations is assessed by associating the correlation coefficients (*R*^2^).  Table 4 displays the equilibrium concentration, *C*_e_, adsorption capacity, *q*_e_, of Pb ions at varying temperatures, and the Pb concentration (initial) obtained using biochars synthesized from peanut shells.

The Langmuir and Freundlich expressions [30, 31] are displayed in equations (3) and (4).

(3) $$\begin{array}{c} q_{e}=\frac{q_{\text{max }}bC_{e}}{1+bC_{e}}, \end{array}$$

where *q*_e_ is the quantity of Pb ions adsorbed/unit weight at equilibrium (mg/g). *q*_max_ is the peak adsorption (mg/g), *C*_*e*_ is the equilibrium of Pb ion concentration (mg/L) and b, the Langmuir constant, is associated with the bonding energy of Pb ion (l/mg).

The adsorption on the multilayer surface is presented by the Freundlich expression equation (4).

(4) $$\begin{array}{c} q_{e}=K_{f\;}C_{e}^{\frac{1}{n}}, \end{array}$$

where *K*_*f* _ (mg/g (1 m/g)^1/n^ and *n* are Freundlich constant indicators of adsorption rate and intensity, respectively.

The Temkin expression in linear form is presented in (5), where A is the Temkin isotherm equilibrium binding constant (L/g) and B is associated with heat from a chemical reaction (adsorption) during the process [32].

(5) $$\begin{array}{c} q_{e}=B\;\ln\;A+B\;\ln\;C_{e}. \end{array}$$

The linear isotherm graphs *C*_e_/*q*_e_ vs. *C*_e_, lnq_e_ vs. lnC_e_, and *q*_e_ vs. lnC_e_ were produced using the experimental data for the Langmuir, Freundlich, and Temkin isotherm expressions. The expression parameters determined from these charts are reported in Table 5. The *R*^2^ values of 1, which indicate the feasibility of the experimental information, are displayed in Table 6. Considering the Langmuir, Freundlich, and Temkin coefficients, the Freundlich coefficient is seen to provide better results than the other two models. The composite adsorbents are generally constituted from materials of similar chemical nature to tailor products of desired quality.

The Freundlich isotherm depends on heterogeneous adsorption zones on the adsorbing surface, with 1/*n* as the function of adsorbing force. It explains the chemical interaction between adsorbate and adsorbent. The value of 1/*n* < 1 indicates the chemical adsorption process, whereas 1/*n* > 1 indicates the process is physical [33]. From Table 7, it is observed that the process is physical. Moreover, the increase in the *K*_f_ with the rise in temperature shows that absorptivity rises with the rise in temperature.

## 5. Conclusion

Biochars obtained from peanut shells and agricultural wastes have been shown to be an effective method to remove Pb from industrial effluent. The use of higher pH resulted in the precipitation phenomenon during the process.

- The increasing temperature was observed during increased chemical reactivity between biochars and contaminant solutions, which resulted in increased adsorption and suggested that the adsorption process was endothermic.
- The reverse trend in adsorption rate was observed concerning the concentration of Pb ions, which is credited to the molecular interactions at the active zones. The high adsorbent dosage is recommended as it can enhance the chemical reactions between the adsorbate and adsorbent to increase the adsorption rate.
- A similar trend was also observed concerning the rise in the mixing speed, as higher mixing speeds produced higher active zones for adsorption. The increase in the contact time was observed to influence the concentration, enhancing the adsorption rate.
- The adsorption kinetics study showed that the synthesized biochars were chemically stable to adsorb the Pb ions onto the surface.

## Figures and Tables

**Table 1:** Proximate and ultimate test of peanut shell samples.

*Proximate test, wt %*				*Ultimate analysis, wt %*					Heating value MJ/Kg
Moisture	Volatile content	Ash content	Fixed carbon	C	H	N	S	O^*∗*^	
2	77	1.95	19.05	47.18	6.1	1.18	1.51	44.03	16

**Table 2:** BET analysis.

BET surface area, m^2^/g	
Temperature°C	Biochar (peanut shell)
500	4.398
600	91.562
700	203.978
800	119.855

**Table 3:** Influence of contact time on adsorption.

C	*q *_*e*_ (mg/g)	Time for equilibrium, min
20	4.2	50
30	7.4	100
40	8.2	150
50	9.7	200

**Table 4:** *C *_O_, ppm, and *q*_e_, mg/g values obtained at varying concentration and temperatures.

*C *_O_, ppm	*T* = 30°C		*T* = 40°C		*T* = 50°C	
	*q *_e_	*C *_*e*_	*q *_e_	*C *_*e*_	*q *_e_	*C *_*e*_
20	3.97	3.39	4.22	3.01	3.58	4.1
30	11.94	6.21	13.59	6.54	10.56	6.72
40	15.79	8.42	16.17	7.88	17.73	8.59
50	22.55	9.24	23.91	8.95	21.62	8.44

**Table 5:** Langmuir isotherms for Pb ions adsorption.

T, °C	*R * ^2^	*q *_max_, mg/g	*K *_L_, L/mg	q_exp_, mg/g
30	0.991	10.98	0.097	3.42
40	0.991	11.25	0.121	3.54
50	0.991	12.82	0.157	3.69

**Table 6:** Freundlich isotherms for Pb ions adsorption.

T, °C	*R * ^2^	*K *_f_, mg/g (L/mg)^1/n^	N, g/L	1/*n*
30	1	1.689	2.16	0.464
40	1	1.921	2.07	0.471
50	1	2.585	2.24	0.484

**Table 7:** Temkin parameters for Pb ions adsorption.

T, °C	*R * ^2^	A	B
30	0.992	1.168	2.422
40	0.992	1.051	2.875
50	0.992	1.543	2.881

## Data Availability

The data used to support the findings of this study are included in the article. Should further data or information be required, these are available from the corresponding author upon request.
